# A dataset of cetacean occurrences in the Eastern North Atlantic

**DOI:** 10.1038/s41597-019-0187-2

**Published:** 2019-09-24

**Authors:** Ana M. Correia, Miguel Gandra, Marcos Liberal, Raul Valente, Ágatha Gil, Massimiliano Rosso, Graham J. Pierce, Isabel Sousa-Pinto

**Affiliations:** 1 Interdisciplinary Centre of Marine and Environmental Research (CIIMAR), 4450-208 Matosinhos, Portugal; 2 Department of Biology, Faculty of Sciences, University of Porto (FCUP), 4169-007 Porto, Portugal; 3 Centre of Marine Sciences (CCMAR), University of the Algarve, Campus de Gambelas, 8005-139 Faro, Portugal; 4 Fraunhofer AICOS, 4200-135 Porto, Portugal; 5 CIMA Research Foundation, 17100 Savona, Italy; 6 Instituto de Investigacións Mariñas (CSIC), 36208 Vigo, Pontevedra, Spain; 7 Oceanlab, University of Aberdeen, Aberdeen, AB41 6AA United Kingdom; 8 CESAM and Department of Biology, University of Aveiro, 3810-193 Aveiro, Portugal

**Keywords:** Biodiversity, Ecosystem ecology

## Abstract

The CETUS project is a cetacean monitoring program that takes advantage of cargo ships to undertake survey routes between Continental Portugal, Macaronesian archipelagos and West Africa. From 2012 to 2017, over 50 volunteers participated in the program, actively surveying more than 124.000 km, mostly beyond national jurisdictions in the high seas, for which little or no previous data existed. In total, the collection comprises 3058 georeferenced transect lines and 8913 positions, which are associated with 2833 cetacean sightings, 362 occurrences of other pelagic megafauna, 5260 estimates of marine traffic and 8887 weather observations. This dataset may provide new insights into the distribution of marine mammals in the Eastern North Atlantic and was published following the OBIS-ENV-DATA format (with the most recent biodiversity data standards at the time of writing). Consequently, it may serve as a model for similar visual line transect data collections yet to be published.

## Abstract (summary)

**Table:** 

Measurement(s)	occurrence • watercraft • weather
Technology Type(s)	visual observation method
Factor Type(s)	cruise • sampling effort • sampling date • geographic location
Sample Characteristic - Organism	Cetacea
Sample Characteristic - Environment	ocean biome
Sample Characteristic - Location	North Atlantic Ocean

Machine-accessible metadata file describing the reported data: 10.6084/m9.figshare.9758591

## Background & Summary

The assessment of the distribution of pelagic apex predators is one of the research priorities for marine management and conservation as these predators are frequently key species in marine ecosystems^1^. However, given their occurrence in the open ocean and their transboundary movements, assessing their distribution is logistically challenging and, consequently, the high seas generally remain poorly surveyed^2–4^. Moreover, the few apex predator occurrences recorded in the open ocean are mostly opportunistic, lacking associated data such as search effort, weather conditions or the presence of marine traffic^5^. At the time of writing, 691 datasets containing 772827 records on cetacean occurrence were compiled in the OBIS-SEAMAP portal (Ocean Biogeographic Information System Spatial Ecological Analysis of Megavertebrate Populations; http://seamap.env.duke.edu). However, until 2016, only 5% of all the marine mammal records were from areas beyond national jurisdiction (i.e., beyond the ~200 nautical mile limit of National Economic Exclusive Zones - EEZ) and nearly 35% of all observations were within the United States EEZ. Moreover, occurrences were also unevenly distributed among species, with few or no records for nearly 26% of cetacean species^6^. This reflects the heterogeneity in sampling effort and the limitations imposed by logistic, economic and weather constraints.

**Fig. 1:**
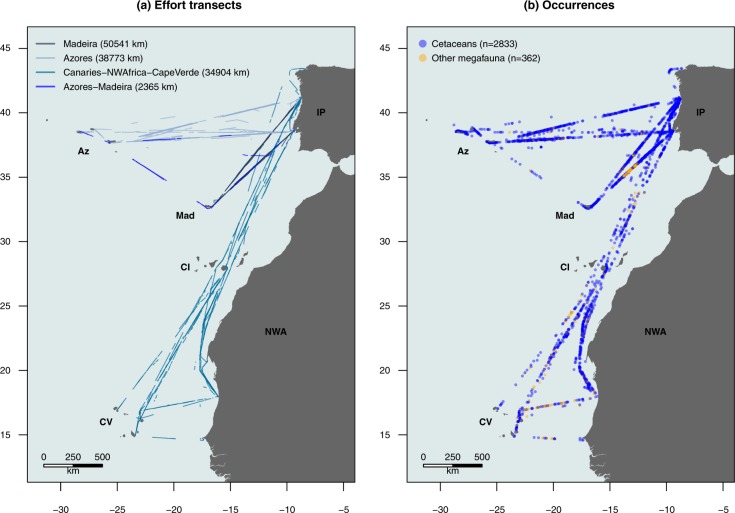
Study area with the on-effort transects and positions included in the dataset. On-effort transects (**a**); Occurrences of cetaceans and other pelagic megafauna (**b**). IP – Iberian Peninsula; NWA – Northwest Africa; Az – Azores; Mad – Madeira; CI – Canary Islands; CV – Cape Verde. Map coordinates are presented in the decimal format.

The CETUS project aims to address these gaps and to study the distribution, habitat characteristics and diversity of cetaceans in the Eastern North Atlantic, with a focus on less surveyed areas, in particular the high seas. CETUS is a cetacean monitoring program that started in 2012, led by the Interdisciplinary Centre for Marine and Environmental Research (CIIMAR). Through a partnership with TRANSINSULAR, a Portuguese company for maritime transport, marine mammal observers (MMOs) board on cargo ships on routes between Continental Portugal, Macaronesian archipelagos and West Africa, to provide new insights into the distribution and occurrence of cetaceans in the Eastern North Atlantic. Use of these so-called “platforms of opportunity” enables the sampling of large geographical areas during extended periods of time at relatively low cost and they are therefore widely used in situations where it is difficult to mount dedicated marine campaigns^7–14^. In addition to the presence of cetacean species and the occurrence of other pelagic megafauna, data on survey effort, weather conditions and marine traffic, among other variables, were collected during the surveys. In total, from 2012 to 2017, observers boarded 430 trips (a trip being a journey from one port to another), surveyed more than 124 000 km on-effort, and registered 8913 positions associated with cetacean sightings (2833 records), other pelagic megafauna occurrences (362 records), marine traffic (5260 records) and weather conditions (8887 records) (Fig. 1).

Part of the information available in the dataset^15^ presented here was already used for developing cetacean habitat models^9,16^, to study migratory movements of baleen whales^17^ and to assess their abundance in the North of Continental Portugal^18^. The monitoring program is still on-going and collecting data with the aim of building the first long-term, wide-range, open-source dataset on cetacean occurrence and distribution in the Eastern North Atlantic. This will allow the study of species distribution patterns, trends in relative abundance, migratory routes and basin-scale habitat use, and will provide important baseline data available to decision-makers and for future management and conservation initiatives.

## Methods

### Geographic location

The dynamic oceanographic processes and complex topographic structures in the Eastern North Atlantic result in a wide variety of habitats which, in turn, support high levels of marine biodiversity^19–21^. Between 2012 and 2017, four different routes were monitored under the CETUS project, all starting in Continental Portugal: Portugal - Madeira (from 2012), Portugal - Azores (from 2014), Portugal - Cape Verde (with stopovers in Canary Islands, Mauritania and Senegal, from 2015) and Portugal - Azores - Madeira (in 2017). The surveyed area ranged in latitude from the north of the Iberian Peninsula (43.4408°N) to Dakar (14.5748°N); and in longitude from northwest Spain (8.3093°W) to the Azores (31.1475°W) (Fig. 1). From the literature, a total of 17 cetacean species has been recorded along the Continental Portuguese coast^22,23^, 26 in Madeira^8,24^, 28 in the Azores^13,25^, 28 in the Canary Islands^26,27^, 24 in Cape Verde^28,29^ and 36 along the Northwestern African coast^30–33^.

### Field surveys

Since 2012, volunteers have been selected to participate as MMOs in the CETUS surveys. From 2014, this selection has been made through an international call that prioritizes volunteers with previous experience in sea-surveys and in marine mammal identification. Nonetheless, each year the MMO team receives an intensive course on line-transect survey protocol and on marine mammal identification before embarking. Each ship then receives a team of two observers, one of them always being experienced in the CETUS protocol (i.e., someone who has previously participated in CETUS campaigns). Each team stays on-board the same ship and route for, at least, one round trip starting in Continental Portugal (boarding and disembarking in the ports of Leixões-Porto or Lisbon).

The sampling protocol follows the standard for line-transect cetacean surveys^2,9,34^ and is performed from sunrise to sunset. Observers stand in the wings of the navigation bridge (at a height of between 13.5 and 16 metres above sea level, considering maximum draught, and depending on the ship). Sporadically, when weather conditions are unsuitable (i.e., strong winds or moderate rain) but there is still adequate visibility, the monitoring is carried from inside the navigation bridge, to ensure continuation of the survey effort. Each MMO stands on one side of the vessel and they switch every 60 minutes (approximately) to avoid fatigue and data-biases. Moreover, in turns, both take one-hour breaks for meals and two optional rests of up to 40 minutes (one in the morning and another during the afternoon).

Monitoring is performed from the front of the vessel with a field of view of approximately 180°, with each MMO covering 90° (except at mealtimes and resting periods, in which case the lone MMO at the survey stand covers the entire 180° from one of the sides range). Binoculars (Porro Prism marine binoculars, with a compass and a distance scale with seven or eight reticles, 7 × 50 mm) are used for occasional scans and to support species identification and group size estimation. Given the nature of the project with the monitoring being performed from a platform of opportunity, it was not possible to have a second independent observer to validate the data collected. Only two observers were allowed to board on each ship (all performing different routes in different periods) and both would stay at the highest point of observation possible performing the monitoring protocol together in order to guarantee a thorough scan of the 180° ahead.

The route of the ship during sampling and the positions marked by the MMOs are recorded using a tablet with an inbuilt GPS and running the application MyTracks (https://my-tracks.pt.aptoide.com). This application registers, among other variables, the date and time (programmed for GMT + 0), the speed (in m/s) and direction (in °) of the vessel and the GPS coordinates (in decimal format, WGS84 coordinate system). The recommended recording settings are maintained: recording every 10 seconds or 10 metres (whichever the smallest) and with minimum precision of 50 m. These settings were occasionally changed in order to overcome battery life issues. The application, although working efficiently at sea, on rare occasions, generates errors in the date and time recording (with the time going forwards and backwards). This issue demands a careful verification process during data entry. An adaptation of the protocol was made during the first year of the project (2012 campaigns), in which a Garmin GPS (with similar settings) was used, with positions being annotated by hand and later imported into Microsoft Excel spreadsheets. During this year, the use of alternative survey stands (e.g. deck of the ship) was explored. Besides data on cetacean occurrences, information about the weather conditions, marine traffic and the presence of other pelagic megafauna is also collected. To record weather conditions, observers assess sea state (using the Douglas scale), wind speed (using the Beaufort scale), visibility (on a categorical scale of values from 1–10 thus covering visibility ranges from 0 m to more than 50000 m, estimated based on the definition of the horizon line and reference points at a known range, e.g., ships with an AIS system) and the occurrence of rain. This information is registered at the beginning and end of each survey leg (a survey leg being a continuous period of sampling) and every time there are significant changes in the conditions. For the marine traffic, small and big vessels (less than and over 20 m in length), detected with or without binoculars, all around the ship´s position, are registered at the beginning and end of each survey, at every sighting of cetacean species and every hour. For pelagic megafauna other than cetaceans, the data are always collected opportunistically, as sampling effort is dedicated uniquely to cetacean species. In these cases, only taxonomic information and the number of individuals (as well as optional comments about the sighting, e.g., animal behaviours, presence of calves or others) are registered. Whenever MMOs cannot gain access to the survey stand (e.g. during safety drills, manoeuvres) or when weather conditions are unfavourable for cetacean monitoring (e.g. at Beaufort or Douglas values > 4, visibility < 1 km or heavy rain), the sampling effort stops and any data collected until effort resumes is considered opportunistic (off-effort).

Whenever a cetacean species is sighted, both observers gather on the relevant side of the boat and mark the end of an on-effort transect in order to correctly collect the data on the occurrence. After registering the sighting, a new on-effort transect starts. Species identification is attempted to the species level, although the identity assigned is always at the taxonomic level at which the MMOs are confident of their identification. Angle of the sighting and vessel direction (angle to the bow) is measured with the binoculars’ compass. Then, these measures are used to calculate the horizontal angle in degrees between the ship’s route and the line to the animal or group of animals (bearing). This bearing ranges between 0 and 360 degrees and is measured in a clockwise direction starting from the ship’s heading (i.e., 0 degrees). As the binoculars’ compasses can be unreliable on platforms containing ferrous metals, the vessel heading is also measured using the binoculars’ compass whenever an animal is sighted. Then, this value is compared with the direction of the route as measured using the GPS, to obtain the approximate error of the compass and correct the registered horizontal angle (during data processing). Additionally, MMOs also measure the vertical angle to the animals by using the reticular-scale in the binoculars (horizontal equally-spaced marks inscribed in the lens). This range estimation method, involves placing the uppermost reticule on the horizon and counting down to the sighted animals. Together with an estimated observation height, these measurements can be then used to calculate the approximate distance to the animals (based on simple trigonometry). It is important to note, however, that for these calculations a mean estimate of the height of the eye-level of the observers should be added to the platform height measures supplied in the dataset, since exact observation heights were not kept.

For group size measures, the observers provide an estimate of the minimum, maximum and assumed (best estimate) number of individuals in a sighting. Moreover, whenever possible, information on the heading of the group and its behaviour towards the ship (i.e. approaching, indifferent or avoiding) is also collected.

### Data processing

After collection, all data between 2012 and 2017 obtained from the GPS recordings and stored in CSV files were imported into Microsoft Excel 2016 spreadsheets and processed on a survey-by-survey basis. Data recorded on paper sheets (in 2012), were entered into the spreadsheets by hand. Data-cleaning procedures were carried out throughout, involving, for example, the conversion of variables to the metric system and correcting the bearing to the sighted animals using the estimated error of the binoculars, as mentioned above. After these operations, data were uploaded to a MySQL database (https://www.mysql.com) for permanent storage and to easily perform queries for verification, validation and export of subsets. All records were then imported into ArcGIS (https://www.esri.com) in order to visualize and correct occasional inaccuracies in the coordinates, and to create the on-effort transect lines with the Data Management tool “Points to Line”. The resulting polyline shapefiles were then used to calculate the effort distances in km (using the Mercator projection) and to describe the transects in the “well-known text” (WKT) format.

### Data management and standardization

In order to comply with the current biodiversity data standards and provide as much information as possible, the entire dataset was reprocessed based on the recently developed OBIS-ENV-DATA format^35^. This new structure was designed for sampling-event datasets, and thus enables the capture of much more detailed information than is found in the widespread “occurrence-only” datasets. It allows the inclusion of important details about the nature of the sampling/observation methods, as well as providing the opportunity to record a multiplicity of biological or environmental measurements collected together alongside the occurrence data. Its underlying conceptual model is a star-like schema (Darwin Core Archive), where one core data file is associated with one or more extension data files through common database keys, i.e. ID fields. In the present format, the core data file holds information about the sampling events (i.e. geographic coordinates, date, protocol, etc.). The occurrences (taxonomical information) and biological/environmental measurements are stored in two separate files: the Occurrence and the ExtendedMeasurementOrFact (eMoF) extensions.

**Fig. 2:**
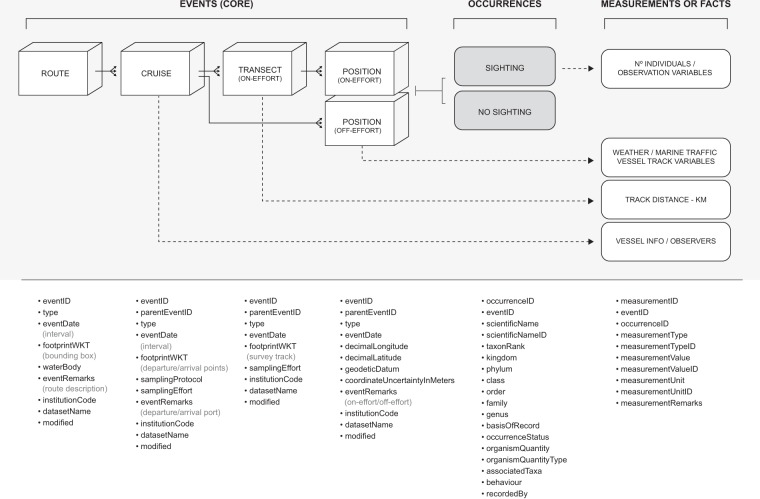
Simplified dataset structure, based on the OBIS-ENV-DATA format. The events’ hierarchy is outlined under the Event Core. The included Darwin Core (DwC, https://dwc.tdwg.org/terms) fields are depicted below each category.

The dataset was exported from the MySQL database and restructured into the appropriate relational format, using custom-written routines in the R environment^36^. All variables were renamed to match the corresponding Darwin Core (DwC) Terms, with a total of 42 controlled terms being used to describe the collected information (for the full list please see Fig. 2).

To reduce data redundancy and facilitate interpretation, an event hierarchy with successive “one-to-many” relationships was created (Fig. 2) comprising four different levels:

1. Here we included events describing the 4 main vessel routes along which surveys were conducted, representing the boundary of the surveyed area in the *footprintWKT* field.
2. Here we described all individual trips, with information about the arrival/departure ports being added to the *eventRemarks* field and the corresponding coordinates being added to the *footprintWKT* as a “MultiPoint” geometry. Additional details about the vessels (name, IMO number and platform height, i.e., distance between the sea-level and the wings of the navigation bridge considering maximum draught), together with the name of the MMOs on-board, were added to the eMoF extension
3. Within each cruise, two types of events were included: the on-effort transects and the off-effort positions (opportunistic sightings or locations where weather/marine traffic measurements were recorded outside effort time). The on-effort transect events include an approximate representation of the survey track in WKT format, as well as sampling dates converted to the ISO 8601 standard. Since most of the transects were approximately linear, the segments were simplified using the Douglas-Peuker algorithm (though the *gSimplify* function available in “rgeos” package^37^; with the objective of decreasing the length of the WKT strings. In some cases, this process resulted in minor differences (mean diff. < 1 km) between the distances originally recorded and those resulting from the simplified segments. In order to preserve the maximum accuracy possible, the effort distances (which are added to both the *samplingEffort* field and the eMoF extension) refer to the original high-resolution trajectories and not to the simplified geometries included in the WKT field. On 14 occasions, only a single GPS position could be logged during the transect, either due to a sighting occurring immediately after the start of the monitoring effort (survey effort stops upon a sighting) or due to rapid changes in weather conditions that hindered the ability of the MMOs to proceed with the survey. In such cases the WKT portrays only the recorded point and the associated track distance was set to zero. Whenever observations were not registered during a transect, we added the coordinates of the centre of the segment to the *decimalLongitude* and *decimalLatitude* terms and calculated the distance between this point and the start or end of the line to include in the *coordinateUncertaintyInMeters* term. This step was required since “absence” records must be associated with specific coordinates in the current repositories.
4. Again, associated with each transect, we added the on-effort positions. Both the on and off-effort positions include detailed geographic coordinates (*decimalLongitude* and *decimalLatitude*) in the EPSG:4326 spatial reference system (WGS84), information about the georeferencing precision (*coordinateUncertaintyInMeters*) and, similarly to the transects, the date in the ISO 8601 format. Information on the vessel speed and heading at each position was added to the eMoF file with the corresponding *eventID* code. To provide improved readability and make the structure more easily perceivable, all event identifiers (*eventID* term) were generated based on the position of each record within the hierarchy, by repeating the preceding parent events on the child records (separated by “:”) and using sequentially assigned numbers, together with a “-OC” suffix to identify positions associated with cetacean sightings and a “-OO” suffix to identify positions associated with other megafauna occurrences.

After completing the structure of the core file, occurrence records were subsequently created by associating each position to the observed taxon and creating absence records whenever cetacean species were not detected. The WoRMS (World Register of Marine Species) webservice was used to validate the scientific names and to extract all the additional taxonomic variables (*scientificNameID*, *taxonRank*, *kingdom*, *phylum*, *class*, *order*, *family* and *genus*). When available, information about taxa associated with the occurrence and their behaviour towards the ship was added to the *associatedTaxa* and *behaviour* DwC terms respectively. Group size estimates (best, maximum and minimum) were added to the eMoF extension, together with information on the bearing to the sighted animals, the number of reticules below horizon and the type of binoculars used, to allow distance-based calculations^38^. However, for these calculations, the user of the dataset has to be aware of the limitations: height of the platform provided is based on maximum draught (which is not always the case), eye-level height will be an average height, possible errors during angle measurements of the observer and of the GPS. The best estimate of the number of individuals was also included in the field *organismQuantity*, for cross-compatibility with other dataset formats.

Finally, all remaining meteorological (weather) and marine traffic measurements were linked to the correspondent events and grouped together in the eMoF file. Whenever possible, the corresponding Unique Resource Identifiers (URIs) were added to the *measurementTypeID* and *measurementUnitID* fields using the NERC Vocabulary Server developed by the British Oceanographic Data Centre (BODC), as recommended for semantic standardization.

## Data Records

The final dataset^15^ was published through the Flanders Marine Institute (VLIZ) IPT portal and distributed by EMODnet and OBIS and can be downloaded as a self-contained file (Darwin Core Archive, DwC-A). The present data descriptor is based on version 1.1 of the dataset.

In total, the dataset contains 12405 events, 9440 occurrences and 86022 measurements or facts, spanning the period from 2012 to 2017 and including nearly 500 days of on-effort surveys. Overall, the collection comprises 44 taxa, 30 of which belong to the infraorder Cetacea (approximately 89% of the sightings). The remaining occurrences, opportunistically registered, consisted mostly of marine turtles (families Cheloniidae and Dermochelyidae, accounting for nearly 9% of the observations) and elasmobranchs (55 sightings, approximately 2% of the observations). Due to occasional visibility limitations inherent to shipboard surveys and in order to ensure a high accuracy of identification, only 46% of the sightings were listed to the species level (Table 1). In total, 30 different species were identified, with more than one third of these being classified as vulnerable, near threatened or endangered by the IUCN Red List (https://www.iucnredlist.org). All records are georeferenced in decimal degrees and include the corresponding dates, resolved to day. Moreover, all on-effort occurrences are associated with the corresponding geospatial transect lines, as well as several observation and weather-related variables to allow end-users to conduct additional analyses.

**Table 1:** Number of occurrences of each recorded taxa.

Taxa	Taxon rank	Number of occurrences
Elasmobranchii	Class	25
Myliobatiformes	Order	1
Cetacea	Infraorder	270
Mysticeti	Superfamily	279
Cheloniidae	Family	173
Delphinidae	Family	750
Istiophoridae	Family	1
Myliobatidae	Family	16
Sphyrnidae	Family	11
Ziphiidae	Family	121
*Globicephala*	Genus	59
*Kogia*	Genus	5
*Morus*	Genus	2
*Thunnus*	Genus	2
*Balaenoptera acutorostrata*	Species	92
*Balaenoptera borealis*	Species	4
*Balaenoptera edeni*	Species	6
*Balaenoptera musculus*	Species	3
*Balaenoptera physalus*	Species	33
*Caretta caretta*	Species	108
*Chelonia mydas*	Species	1
*Delphinus delphis*	Species	394
*Dermochelys coriacea*	Species	3
*Grampus griseus*	Species	8
*Hyperoodon ampullatus*	Species	4
*Lagenodelphis hosei*	Species	1
*Manta birostris*	Species	2
*Megaptera novaeangliae*	Species	9
*Mesoplodon densirostris*	Species	5
*Mola mola*	Species	16
*Monachus monachus*	Species	1
*Orcinus orca*	Species	5
*Peponocephala electra*	Species	4
*Phocoena phocoena*	Species	4
*Physeter macrocephalus*	Species	152
*Pseudorca crassidens*	Species	13
*Stenella attenuata*	Species	8
*Stenella clymene*	Species	16
*Stenella coeruleoalba*	Species	154
*Stenella frontalis*	Species	226
*Stenella longirostris*	Species	6
*Steno bredanensis*	Species	4
*Tursiops truncatus*	Species	134
*Ziphius cavirostris*	Species	64
**TOTAL**	**44 taxa**	**3195 occurrences**

The number of occurrences is presented by taxa recorded to the highest possible level. The table is organized by taxon rank of the records and alphabetically within.

To our knowledge, this is one of the first cetacean survey datasets to be made available in the recent OBIS-ENV-DATA format and thus we believe it can provide a model to be considered when similar visual line transect data collections are assembled in the future.

## Technical Validation

Every year, within CETUS Project, observers that board on the cargo ships receive an intensive training on both the sampling protocol and marine mammals’ identification. Moreover, during selection process, applicants are evaluated and selected according to their previous experience on cetacean identification and fieldwork at sea; and the interest of the observers to participate in CETUS as part of an internship for academic purposes is encouraged to guarantee personal interest in the surveys. Within the boarding team, at least one of the observers has experience in the survey protocol (i.e., boarded before with the CETUS Project). Observers only register the identifications to the taxonomic level they are confident with, hence, the accuracy of the collected data is assured.

During data processing and development of the final dataset, verifications and validations were made at several stages: during the digitalization of the data to the excel files, within the MySQL database, in ArcGIS and in R, after structuring the final dataset. Up to the ArcGIS stage, these verifications were made every year (i.e., after compiling the data for that year). Posteriorly, a thorough validation was undertaken by the members of the research team. This included checking mismatching codes, cross-crossing dates and coordinates across the dataset schema (i.e., route-cruises-segments-positions), confirming segment and effort distances, verifying taxonomy and standardizing nomenclature.
